# Intrinsic Functional Connectivity in the Default Mode Network Differentiates the Combined and Inattentive Attention Deficit Hyperactivity Disorder Types

**DOI:** 10.3389/fnhum.2022.859538

**Published:** 2022-06-09

**Authors:** Jacqueline F. Saad, Kristi R. Griffiths, Michael R. Kohn, Taylor A. Braund, Simon Clarke, Leanne M. Williams, Mayuresh S. Korgaonkar

**Affiliations:** ^1^Brain Dynamics Centre, Westmead Institute for Medical Research, The University of Sydney, Sydney, NSW, Australia; ^2^School of Medicine, Faculty of Medicine and Health, The University of Sydney, Sydney, NSW, Australia; ^3^Centre for Research Into Adolescent’s Health, Department of Adolescent and Young Adult Medicine, Westmead Hospital, Sydney, NSW, Australia; ^4^Black Dog Institute, University of New South Wales, Sydney, NSW, Australia; ^5^School of Psychiatry, University of New South Wales, Sydney, NSW, Australia; ^6^Department of Psychiatry and Behavioral Sciences, Stanford University, Stanford, CA, United States; ^7^Sierra Pacific Mental Illness Research Education and Clinical Center, VA Palo Alto Health Care System, Palo Alto, CA, United States

**Keywords:** ADHD combined, ADHD inattentive, brain functional connectivity, functional connectome, network-based statistics, default mode network

## Abstract

Neuroimaging studies have revealed neurobiological differences in ADHD, particularly studies examining connectivity disruption and anatomical network organization. However, the underlying pathophysiology of ADHD types remains elusive as it is unclear whether dysfunctional network connections characterize the underlying clinical symptoms distinguishing ADHD types. Here, we investigated intrinsic functional network connectivity to identify neural signatures that differentiate the combined (ADHD-C) and inattentive (ADHD-I) presentation types. Applying network-based statistical (NBS) and graph theoretical analysis to task-derived intrinsic connectivity data from completed fMRI scans, we evaluated default mode network (DMN) and whole-brain functional network topology in a cohort of 34 ADHD participants (aged 8–17 years) defined using DSM-IV criteria as predominantly inattentive (ADHD-I) type (*n* = 15) or combined (ADHD-C) type (*n* = 19), and 39 age and gender-matched typically developing controls. ADHD-C were characterized from ADHD-I by reduced network connectivity differences within the DMN. Additionally, reduced connectivity within the DMN was negatively associated with ADHD-RS hyperactivity-impulsivity subscale score. Compared with controls, ADHD-C but not ADHD-I differed by reduced connectivity within the DMN; inter-network connectivity between the DMN and somatomotor networks; the DMN and limbic networks; and between the somatomotor and cingulo-frontoparietal, with ventral attention and dorsal attention networks. However, graph-theoretical measures did not significantly differ between groups. These findings provide insight into the intrinsic networks underlying phenotypic differences between ADHD types. Furthermore, these intrinsic functional connectomic signatures support neurobiological differences underlying clinical variations in ADHD presentations, specifically reduced within and between functional connectivity of the DMN in the ADHD-C type.

## Introduction

The field of brain functional connectomics is rapidly expanding, revealing insights into large-scale brain networks underlying Attention Deficit Hyperactivity Disorder (ADHD) (Cao et al., 2014; Castellanos and Aoki, 2016), a prevailing neurodevelopmental condition with an estimated heritability of 80% (Faraone and Larsson, 2019) and an overall global prevalence of 5.9% (Thapar and Cooper, 2016; Faraone et al., 2021). Inter-regional network organization comprising frontal, parietal, cerebellar, and cortico-striatal thalamic regions (Castellanos and Proal, 2012; Posner et al., 2014) reflect cognitive and emotional processes in ADHD (Bush, 2010; Faraone et al., 2015). While this body of work has highlighted network phenotypic alterations in ADHD associated with its core clinical features, the neurobiological mechanisms underlying ADHD types remain unclear and a gap in the field (Rubia, 2018). Therefore, it is crucial to establish clear neurobiological pathways for ADHD presentation types to improve diagnostic accuracy and understand how these relate to treatment prediction and clinical outcomes (Cao et al., 2014; Saad et al., 2020); knowledge which is imperative to advancing our understanding of ADHD pathophysiology.

Clinically characterized by symptom clusters of inattentiveness, hyperactivity-impulsivity, or both, ADHD presentation types are classified as predominantly inattentive (ADHD-I), predominantly hyperactive-impulsive (ADHD-HI), or combined (ADHD-C) (DSM-V., 2013), respectively. Symptom variation within these clinical presentation types reflects the etiological heterogeneity of ADHD (Luo et al., 2019; Nigg et al., 2020). Studies show inattentive symptoms endure across the lifespan, in contrast to diminishing hyperactive-impulsive symptoms in late adolescence-young adulthood, which infers that ADHD types have differential developmental trajectories (Willcutt et al., 2012; Lee et al., 2016; Sudre et al., 2021). Furthermore, variations in clinical symptoms amongst ADHD types may originate from dysfunctional neural networks associated with inefficient regulation of neurocognitive processes (Cao et al., 2014; Qian et al., 2019; Saad et al., 2020). Adding to this, the idea that a singular mechanism underpins dysfunction or dysregulation has evolved. More recent accounts include a multifactor framework encompassing several large-scale brain network pathways that underpin the clinical symptoms of ADHD and its types (Castellanos and Aoki, 2016; Stevens et al., 2018; Pruim et al., 2019). The ability to capture information on large-scale intrinsic functional brain networks using connectomic imaging techniques has shed light on the disorganization of critical neural networks in ADHD. The field has witnessed a proliferation of functional network analyses in the ADHD literature (Cao et al., 2014), utilizing methodologies such as network-based statistics (NBS) and graph-theoretical analysis (Griffiths et al., 2021). This approach may reveal the neural architecture underlying ADHD functional symptoms and shed light on the altered neural network mechanisms that may drive the differences in clinical symptoms associated with ADHD-C and ADHD-I types (Fair et al., 2012; Cao et al., 2014).

Disruptions of intrinsic brain network connectivity and task-based functional connectivity involving the default mode network (DMN), cingulo-opercular, frontoparietal and executive control [referred to as the cingulo-frontoparietal (CFP) attention network in this study], ventral attention network (VAN), and the somatomotor [also known as the somatosensory network (SMN)] are found to characterize ADHD (De La Fuente et al., 2013; Posner et al., 2014; Castellanos and Aoki, 2016; Bos et al., 2017; Stevens et al., 2018; Pruim et al., 2019; Kowalczyk et al., 2021; Lin et al., 2021). Structures of the cingulo-frontoparietal (CFP) attention network include frontostriatal and frontoparietal pathways (Bush, 2010), modulating attention, executive function including working memory, response inhibition, motor control and reward/motivation (Bush, 2010; Lin et al., 2015; Saad et al., 2020). Recent meta-analyses of large-scale brain networks in ADHD identify critical networks in understanding the neural mechanisms that drive ADHD symptoms, particularly highlighting the impact of reduced intra-connectivity between the DMN and cognitive control and limbic and salience networks (Gallo and Posner, 2016; Gao et al., 2019; Sutcubasi et al., 2020; Cortese et al., 2021). Additionally, the VAN (also referred to as the salience network) engagement with the DMN and CFP network are thought to underpin symptoms observed in this condition (Menon, 2011; Gao et al., 2019; Pruim et al., 2019).

Findings from functional imaging studies have also provided a window into the neural pathways underlying the clinical presentations of ADHD, irrespective of shared core clinical symptoms (De La Fuente et al., 2013; Cao et al., 2014). While reports on direct comparisons of task-based connectivity differences between ADHD types have been limited (Saad et al., 2020; Kowalczyk et al., 2021), task-based fMRI studies have shown differences in the CFP attentional network and VAN in ADHD-I (Solanto et al., 2009; Orinstein and Stevens, 2014), occipital-parietal and visual attention networks (VN) in ADHD-C (Solanto et al., 2009) relative to each other. Correspondingly, resting-state studies have identified dysfunctional connectivity within and between large-scale functional networks proposed to underlie the behavioral and neurocognitive characteristics of these two clinical ADHD types [recently reviewed in Posner et al. (2014), Gallo and Posner (2016), Gao et al. (2019)]. In addition, we recently reviewed intrinsic functional connectivity studies examining differences between ADHD types (Saad et al., 2020), summarizing primarily altered connectivity and network disorganization of the DMN in ADHD-C, the CFP attention network in ADHD-I, with shared disruptions in the sensorimotor network. More recently, studies have found mixed findings of atypical connectivity within the DMN (Qian et al., 2019; Zhang et al., 2020), hypoconnectivity within the dorsal attention network (DAN), hyper-connectivity of the VN, cerebellum and limbic networks (Qian et al., 2019) characterizing ADHD-C from ADHD-I. Significantly, the research explicitly examining intrinsic functional connectivity underlying the two ADHD clinical presentation types is insubstantial. Therein lies the opportunity to investigate whether intrinsic neural characteristics underlying the functional deficits of ADHD may differentiate the ADHD types.

The role of the DMN in ADHD pathophysiology has been at the forefront of ADHD research, reporting increased and decreased connectivity differences between ADHD types. The DMN is primarily activated during resting states associated with internally focused tasks, goal-directed activity, and distractibility (Mohan et al., 2016), serving as a state-based regulatory mechanism (Raichle, 2015). There is an inverse relationship between the DMN and CFP, specifically with downregulation of DMN activity and increased activity in the CFP during cognitive demands. As such, this highlights the critical role of the DMN in modulating attentional performance and response inhibition (Bush, 2010; Sripada et al., 2014a). There is some evidence that ineffective suppression of DMN activity during task demands interferes with networks such as the CFP and VAN, resulting in suboptimal cognitive performance characteristic of the clinical deficits in ADHD (Liddle et al., 2011; Sripada et al., 2014a; Carmona et al., 2015; Gallo and Posner, 2016; Silk et al., 2017), particularly the ADHD-C type (Fair et al., 2012). Studies have also found functional connectivity between anterior and posterior components (subdivisions) of the DMN (Rubia, 2018; Cortese et al., 2021) in ADHD types to differ with implications for pathway-specific findings underlying functional symptoms (Fair et al., 2012; Qian et al., 2019). Despite these encouraging findings, no resting-state intrinsic connectivity studies thus far have conducted a comprehensive connectome-wide inspection of whole-brain intrinsic connectivity in the ADHD-C and ADHD-I types incorporating NBS (Zalesky et al., 2010) and graph-theoretic analysis (Bullmore and Sporns, 2009). Findings from previous studies have employed methodologies reliant upon inspection of *a priori* specific networks and seed selection, predominantly in cohorts inclusive of all ADHD types or the non-disclosure of ADHD type (Castellanos and Aoki, 2016; Samea et al., 2019), which may bias results and limit the discovery of connectivity patterns differentiating the ADHD types (Qian et al., 2019; Zhou et al., 2019). Given that the DMN and CFP networks have received substantial coverage, research must be examined from a whole-brain connectome perspective, including within and between network connectivity related to the other large-scale intrinsic networks of the brain (Gao et al., 2019). Furthermore, findings of alterations in functional connectivity are equivocal; thus, evidence for these connectivity differences in ADHD types is constrained by the paucity of studies available in the field (see meta-analyses Samea et al., 2019; Cortese et al., 2021; Pereira-Sanchez and Castellanos, 2021).

To address this gap, we extracted task-derived rs-fMRI data acquired from children and adolescents with ADHD to comprehensively evaluate both whole-brain and DMN functional network topology utilizing network-based statistical and graph-theoretical analytic approaches. Our first goal was to investigate whether large-scale brain networks distinguished ADHD-C and ADHD-I types. Based on previous evidence, we expected the DMN to characterize ADHD-C and cingulo-frontoparietal networks associated with ADHD-I, with an overlap in sensorimotor connectivity (Bush, 2010; Fair et al., 2012; Cao et al., 2014; Faraone et al., 2015; Gallo and Posner, 2016; Qian et al., 2019). In this same cohort, we have previously demonstrated differences in graph properties of structural volume covariance networks in ADHD from controls (Griffiths et al., 2016); and between the two ADHD types and alterations within the DMN (Saad et al., 2017). With structural covariance networks proposed to parallel functional connectivity (Irimia and Van Horn, 2013), we expected functional connectivity differences in graph topological measures between the two subtypes should also be present.

## Materials and Methods

### Participant Characteristics and the Study Protocol

Attention Deficit Hyperactivity Disorder participants were recruited and tested as part of the International Study to Predict Optimized Treatment in ADHD (iSPOT-A). The iSPOT-A inclusion/exclusion criteria protocols outlining participant recruitment, diagnostic measures and procedures for iSPOT-A has been previously published (Elliott et al., 2014). In addition, healthy control participants were tested as part of a separate study, NHMRC funded project grant (APP1008080) Limbic Maturational Changes in Young Adulthood study (LIMCA) (Grieve et al., 2011) awarded to Korgaonkar and Williams. The iSPOT-A and the LIMCA study applied the same study protocols and procedures, including scanner hardware, previously described (Grieve et al., 2011; Cai et al., 2019).

Task functional magnetic resonance imaging (fMRI) data collected at Westmead Hospital, Sydney, NSW, Australia, as part of the baseline fMRI scans from the two studies were available for 34 participants with ADHD (mean age 13.48 ± 2.50; range 8–17 years) and 39 age and gender-matched healthy control participants (mean age 13.23 ± 2.49; range 8–17 years). Confirmation of ADHD diagnosis (DSM-IV criteria), subtype (i.e., presentation type; DSM-V), was measured by the Mini International Neuropsychiatric Interview (MINI Kid) (Sheehan et al., 2010), the Attention Deficit-Hyperactivity Disorder Rating Scale (ADHD-RS IV) (DuPaul et al., 1998) to assess symptom severity (requires a score of >1 on six or more subscale items on the Inattentive and Hyperactive-Impulsive subscales or both) and the Conner’s Parent Rating Scale-Revised: Long Version (CPRS-LV) (Elliott et al., 2014). Of the 34 ADHD participants, 19 met the criteria for ADHD-C type (mean age 13.39 ± 2.47; 4 females), while 15 met ADHD-I type criteria (mean age 14.25 ± 2.56; 4 females). Participants from the ADHD-C group (*n* = 5) and ADHD-I group (*n* = 2) had a diagnosis of oppositional defiant disorder (ODD). All (*n* = 34) ADHD participants were medication-free at testing; 16 were medication naïve; 18 treatment-experienced withdrew from methylphenidate for at least five half-lives. All participants were fluent in English, with no history of brain injury or significant medical conditions affecting brain function (e.g., epilepsy) or contraindications for MRI. All participants, their guardians, or both, were provided with a written informed consent form to participate in the research, per National Health and Medical Research Council (NHMRC) guidelines and institutional review board ethical guidelines (Western Sydney Local Health District Human Research Ethics Committee).

### Functional Magnetic Resonance Imaging Image Acquisition and Pre-processing

Structural and functional magnetic resonance images (MRI) were acquired by the Department of Radiology, Westmead Hospital, Sydney, NSW, Australia, on a 3T GE Signa HDx scanner (GE Healthcare, Milwaukee, WI, United States) using an eight-channel phased-array head coil. The image acquisition and pre-processing details have been previously described (Korgaonkar et al., 2013, 2020) and are briefly described here. Echo planar imaging (EPI) sequence was utilized for the fMRI data collection (repetition time = 2.5 s; echo time = 40 ms; matrix = 64 × 64; field of view = 24 cm; flip angle = 90°; slice thickness = 3 mm with no slice gap; 43 axial slices). At the start of each acquisition, three dummy scans were acquired, and for each task protocol, a collection of 120 volumes with a total scan time of 5 min and 8 s (Korgaonkar et al., 2013). MRI task activation data involved five fMRI tasks, of which task derived resting-state data were estimated for the intrinsic functional connectivity analysis. The five functional MRI tasks completed by participants involved the Go-NoGo (response inhibition), auditory oddball, facial emotion processing (non-conscious and conscious) and working memory (n-back) (previously described Korgaonkar et al., 2014, 2020). Removal of task-related variance from each task was modeled utilizing the general linear model framework, saving the residuals from each analysis (Korgaonkar et al., 2014). These residuals represented task-free resting data which were then concatenated across the five fMRI tasks and were used for connectivity analyses as described below.

Three-dimensional (3-D) T1-weighted structural magnetic resonance images were acquired in the sagittal plane using a 3D SPGR sequence (repetition time = 8.3 ms; echo time = 3.2 ms; flip angle = 11 degrees; inversion time = 500 ms; matrix = 256 × 256; NEX = 1; ASSET = 1.5; Frequency direction: S/I; slice thickness = 1 mm with no slice gap, 180 slices; with an in-plane resolution of 1 mm × 1 mm resulting in isotropic voxels). The sequence was used to normalize the functional data to MNI standard space.

### Task Derived Resting State Data Analysis and Generation of Functional Connectomes

#### Pre-processing of Connectivity Data

Our previous work has documented the pre-processing steps for structural and functional images performed using the Statistical Parametric Mapping software (SPM8) package in MATLAB and VBM8 toolbox (Korgaonkar et al., 2013, 2020; Saad et al., 2017). In addition to removal of task-related variances from the fMRI data, data volumes associated with high significant movement (framewise displacement from one-time point to the next) or changes in blood-oxygenation-level-dependent (BOLD) signal intensity (as indexed by the spatial standard deviation of successive difference images (DVARS) (Power et al., 2012) were censored (temporally masked) reducing the influence of motion and related artifacts (Power et al., 2012; Ciric et al., 2017; Goldstein-Piekarski et al., 2018). Based on Power et al. (2014), framewise displacement was calculated as the sum of the absolute values of the differentiated realignment estimates. Established thresholds of framewise displacement equal to or greater than 0.3 mm and scaled signal intensity differences greater than 10 were used for volume censoring (Power et al., 2012, 2014; Ciric et al., 2017), which were then examined using the TSDiffAna toolbox^1^ and in-house scripts. Creation of a temporal mask for each censored volume used as regressors of no interest in the first level statistical models (Power et al., 2012, 2014). To reduce movement-related artifacts, a total of four temporal masks were created for each movement spike, including an additional volume before and two volumes after the movement spike, as movement-related artifacts can impact volumes acquired before and several seconds after a movement spike (Power et al., 2014). Additionally, the temporal mask included the censored volume and one subsequent volume with signal change spikes. For each of the five fMRI tasks, the average signal time course from cerebrospinal fluid (CSF) and white matter (WM) masks were extracted to regress out the physiological noise covariates, as well as the temporal masks derived from the volume censoring described above and motion effects using the Volterra expansion of the Friston 24 (Friston et al., 1996) proposed realignment parameters (Korgaonkar et al., 2013, 2020). Using a regression model, voxel-wise BOLD time series was analyzed against the task covariates included in the design matrix as nuisance regressors (WM and CSF), as the residual images of this model to estimate the intrinsic functional connectivity signal. Temporal band-pass filtering with cut-off frequency values of 0.009–0.08 Hz, was applied to the time series following the denoising procedure (Korgaonkar et al., 2013).

#### Generation of Whole Brain and Default Mode Network Connectivity Matrices

Using ROI-ROI connectivity estimation, functional connectomes were generated as a correlation matrix (inter-regional connectivity) for the whole-brain and the default mode network (DMN). The automated anatomical labeling (AAL) 116 atlas (Tzourio-Mazoyer et al., 2002) defined the parcelation scheme of functional MRI data into 92 cortical and subcortical gray matter regions, derived from combining 90 cortical and subcortical regions and 26 cerebellar regions averaged into two hemispheres. That is, of the 26 cerebellar regions, eighteen cerebellar subregions were averaged into two regions as the left and right cerebellum (8 vermis regions not included due to lack of lateralization). Thus, the two cerebellar regions together with the 90 cortical and subcortical regions resulted in a total of 92 regions. The automated anatomical labeling (AAL) 116 atlas (Tzourio-Mazoyer et al., 2002) defined the parcelation scheme of functional MRI data into 92 cortical and subcortical gray matter regions, derived from combining 90 cortical and subcortical regions and 26 cerebellar regions averaged into two hemispheres. That is, of the 26 cerebellar regions, eighteen cerebellar subregions were averaged into two regions as the left and right cerebellum (8 vermis regions not included due to lack of lateralization). Thus, the two cerebellar regions together with the 90 cortical and subcortical regions resulted in a total of 92 regions. Intrinsic functional time series were extracted from each participant for each AAL region, and inter-regional correlations were used to create a 92 × 92 inter-regional functional connectivity matrix for every individual. Correlation coefficients were transformed into *z* scores using Fisher’s Z-transformation. The DMN connectivity matrix was derived using an available DMN ROI mask used in the field (Fair et al., 2008) and our previous structural MRI study (Saad et al., 2017). The DMN ROI mask comprised the following thirteen regions: the ventromedial prefrontal cortex (vMPFC), anterior medial prefrontal cortex (aMPFC), posterior cingulate cortex (PCC), lateral parietal cortex, bilateral: superior frontal cortex, inferior temporal cortex, parahippocampal gyrus, cerebellar tonsils, and the retrosplenial cortex (RSC). ROI masks were created using a sphere with an 8 mm radius centered on MNI coordinates.

### Network-Based Statistics

Network-based statistical analyses (NBS) (Zalesky et al., 2010) were applied to examine differences in intrinsic functional connectivity between the two ADHD types and relative to the control group using the interregional 92 × 92 connectivity matrix for the whole brain and 13 × 13 matrix for the DMN. A key advantage in applying NBS is its power in evaluating interconnected subnetworks and its ability to minimize the extensive multiple comparisons that arise when evaluating connectomic data (Zalesky et al., 2010). First, we performed a two-sample *t*-test at every inter-regional connection applying a test statistic (*t*) threshold of 3 as a primary component-forming threshold corresponding to an uncorrected level of *p* < 0.001. For the DMN, we used a less stringent t threshold of 2 corresponding to an uncorrected level of *p* < 0.05, considering that we had fewer connections for the DMN matrix for this analysis. This was done to identify any connected components, and next, the size of each component was evaluated with the family-wise error (FWE) corrected *p*-value of 0.05 used with 5,000 permutations for both the whole brain and default mode network analyses.

To help interpret the connectivity findings in intrinsic brain network definitions, we used the Yeo 7 network atlas (Yeo et al., 2011), a functional parcelation based on resting-state connectivity data classified into seven networks (Qian et al., 2019). These seven networks include the DMN, CFP (i.e., frontoparietal, FPN), limbic, DAN, VAN (i.e., salience network, SN), somatomotor (i.e., SSN), and visual networks (Yeo et al., 2011).

### Graph Theory Analyses

Using the Brain Connectivity Toolbox, graph-theoretical analyses were performed on the whole brain and DMN connectivity matrices^2^ (Rubinov and Sporns, 2010). Two measures of global topological properties of the brain were estimated: (i) characteristic path length (i.e., the mean number of connections on the shortest path between any two regions in the network) and (ii) clustering coefficient, which quantifies the probability that two nodes connected to an index node are also connected to each other. We also examined regional nodal degree characteristics (i.e., number of connections a node has with the rest of the network). Only the global network properties were examined for the default mode network. The matrices were thresholded at a range of network densities in 0.01 steps (Dmin: 0.03: 0.50) to compare network properties between the groups and avoid biases associated with using a single threshold. For both the global assessment of the two different metrics and regional network measures to account for the number of comparisons across the 92 brain regions, we applied the Benjamini-Hochberg (Benjamini et al., 2001) method to obtain the false discovery rate (FDR) corrected *p* < 0.05 for statistical evaluation.

Differences in demographic and clinical characteristics were analyzed using analysis of variance (ANOVA), with corrected *p*-values adjusted to *q*-values using the false discovery rate (FDR) via the Benjamini-Hochberg (Benjamini et al., 2001) approach. In addition, we employed correlation analyses to assess associations between significant network connections identified from the NBS analyses and the graph measures with ADHD-RS clinical symptom scores. The visualization of NBS results was produced using the BrainNet Viewer (Xia et al., 2013) toolbox^3^ via MATLAB.

## Results

Table 1 summarizes clinical and demographic characteristics for the two ADHD groups and control participants. The three groups did not significantly differ in age or gender. Medication treatment history and the ADHD-RS-IV (inattentive symptom items) sum of items 1–9 did not significantly differ between subtypes. ADHD-C type significantly differed from ADHD-I on the sum of items 10–18 (hyperactive-impulsive symptom items) and total item scores of the ADHD-RS-IV, typically associated with the ADHD-C type criteria and severity (*p* < 0.01). Of the ADHD-I type participants, one participant qualified for seven items out of 9 hyperactive-impulsive subscale items, five items (*n* = 1), four items (*n* = 2), three items (*n* = 1), two items (*n* = 1) and ≤1 item (*n* = 9). Head motion censoring data showed no significant differences between the ADHD subtypes or the ADHD subtypes and controls.

**TABLE 1:** Participant demographic and clinical characteristics.

	ADHD-C (*n* = 19)	ADHD-I (*n* = 15)	Controls (*n* = 39)		
	Mean ± SD	Mean ± SD	Mean ± SD	*p*	Cohen’s *d*
Gender, female, *n* (%)	4 (21%)	4 (27%)	16 (41%)	0.329	
Age, years	13.39 ± 2.47	14.25 ± 2.56	13.23 ± 2.49	0.406	
Education, years (range)	6.37 ± 2.59 (6–18)	6.73 ± 2.81 (6–18)	7.64 ± 3.00 (6–18)	0.245	
Head motion volume censoring	85.26 ± 55.76	62.67 ± 44.39	69.59 ± 60.68	0.470	
ADHD-RS IV Int Subscales	20.84 ± 3.42	21.60 ± 3.94	−	0.553	−0.207
ADHD-RS IV Hyp-Imp Subscales	19.32 ± 3.28*	7.47 ± 4.66	−	<0.001*	**3.004**
ADHD-RS IV Total Item score	40.16 ± 5.36*	29.07 ± 5.43	−	<0.001*	**2.058**
Medication Naïve	10 (53%)	6 (40%)	−	0.479	
Oppositional defiant disorder, *n* (%)	5 (26%)	2 (13%)	−	0.368	

Significant differences between the ADHD-C and ADHD-I subtypes at p < 0.05 are highlighted in bold. *p < 0.05 for comparisons between the ADHD-C and ADHD-I types. ADHD-C, ADHD combined presentation; ADHD-I, ADHD predominantly inattentive presentation; ADHD-RS IV, attention deficit hyperactivity disorder rating scales-version 4; ADHD-RS IV Int Subscale; inattentive subscale score, ADHD-RS IV Hyp-Imp subscales score; hyperactivity-impulsivity subscales score.

### Network-Based Statistics

#### Network Functional Connectivity Differences in ADHD-C but Not ADHD-I, Compared With Controls

**FIGURE 1:**
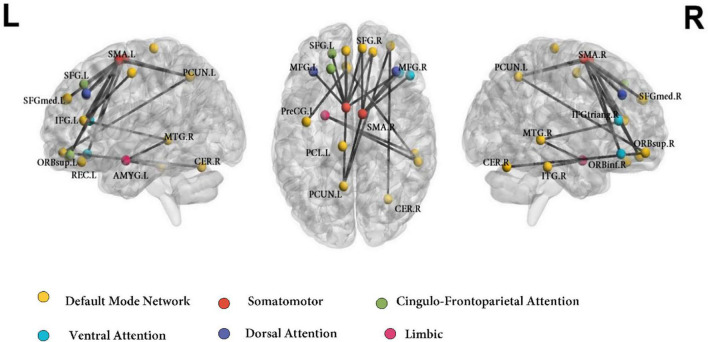
Functional connectivity networks differentiating ADHD-C and Control groups using the AAL atlas. Colors of the AAL nodes represent the intrinsic brain networks that they belong to from mapping functional parcelation atlas onto Yeo’s seven resting-state network atlas. DMN, default mode network; CFP, cingulo-frontoparietal network; SSN, somatomotor; VAN, ventral attention network; DAN, dorsal attention network; L, left; R, right. ORBinf.R, inferior frontal gyrus, orbital part (Right); MFG.L, middle frontal gyrus (Left); SFGdor.L, superior frontal gyrus, dorsolateral (Left); SFGmed.L, superior frontal gyrus, medial (Left); SFGmed.R, superior frontal gyrus, medial (Right); ORBsup.L, superior frontal gyrus, orbital part (Left); ORBsup.R, superior frontal gyrus, orbital part (Right); IFGtriang.R, inferior frontal gyrus, triangular part (Right); ORBmid.R, superior frontal gyrus, medial orbital (Right); MFG.R, middle frontal gyrus (Right); PreCG.L, precentral gyrus (Left); SMA.L, supplementary motor area (Left); SMA.R, supplementary motor area (Right); ACG.L, anterior cingulate gyrus (Left); AMYG.L, amygdala (Left); REC.L, gyrus rectus (Left); PCUN.L, precuneus (Left); CER.R, cerebellum (Right); MTG.R, middle temporal gyrus (Right); ITG.R, inferior temporal gyrus (Right); PCL.L, paracentral lobule (Left).

The whole-brain NBS analysis identified a connectomic signature comprising 24 inter-regional connections across 21 nodes between ADHD-C and controls (ADHD-C < controls; Figure 1 and Table 2; *p* = 0.035 corrected for multiple comparisons). This connectomic signature was characterized by: (1) reduced inter-network intrinsic functional connectivity: (a) between regions of the somatomotor and DMN; (b) between regions of the somatomotor, cingulo-frontoparietal, ventral attention and dorsal attention networks; (c) between regions of the DMN, and the limbic networks; and (2) reduced intra-network intrinsic functional connectivity within the DMN. No significant inter-network connectivity differences were identified between ADHD-C and ADHD-I or ADHD-I and controls, corrected for multiple comparisons.

**TABLE 2:** Networks identified to be significantly different between the ADHD-C and control groups using network-based statistical analysis.

Significant network connections (AAL regions)	Intrinsic functional connectivity			

	ADHD-C (*n* = 19)	Controls (*n* = 39)		
	(Mean ± SD)	(Mean ± SD)	*t* and *p*-value	*Cohen’s d*
*Default mode – Default mode*				
L – precentral gyrus to L – anterior cingulum	−0.19 ± 0.14	−0.04 ± 0.18	*t* = 3.15, *p* = 0.002	−*0.889*
L – anterior cingulum to L – paracentral lobule	−0.22 ± 0.14	−0.02 ± 0.19	*t* = 4.07, *p* < 0.001	−*1.132*
R – middle orbitofrontal gyrus to R – inferior temporal gyrus	0.17 ± 0.16	0.39 ± 0.22	*t* = 4.03, *p* < 0.001	−*1.126*
R – middle orbitofrontal gyrus to R – middle temporal gyrus	0.08 ± 0.19	0.25 ± 0.17	*t* = 3.58, *p* = 0.001	−*0.999*
L – gyrus rectus to R – cerebellum	0.05 ± 0.23	0.24 ± 0.23	*t* = 3.05, *p* = 0.003	−*0.855*
L – anterior cingulum to L – gyrus rectus	0.14 ± 0.19	0.31 ± 0.21	*t* = 3.00, *p* = 0.004	−*0.836*
*Default mode – Somatomotor*				
R – inferior temporal gyrus to L – supplementary motor area	0.08 ± 0.18	0.23 ± 0.16	*t* = 3.41, *p* = 0.001	−*0.953*
R – inferior temporal gyrus to R – supplementary motor area	0.07 ± 0.20	0.27 ± 0.15	*t* = 4.11, *p* < 0.001	−*1.151*
L – anterior cingulum to L – supplementary motor area	0.10 ± 0.18	0.28 ± 0.20	*t* = 3.46, *p* = 0.001	−*0.966*
L – medial superior frontal gyrus to L – supplementary motor area	0.19 ± 0.16	0.40 ± 0.22	*t* = 3.67, *p* = 0.001	−*1.020*
R – medial superior frontal gyrus to L – supplementary motor area	0.09 ± 0.16	0.26 ± 0.19	*t* = 3.51, *p* = 0.001	−*0.982*
R – middle orbitofrontal gyrus to R – supplementary motor area	0.04 ± 0.16	0.19 ± 0.18	*t* = 3.02, *p* = 0.004	−*0.853*
R – medial superior frontal gyrus to R – supplementary motor area	0.00 ± 0.14	0.16 ± 0.19	*t* = 3.30, *p* = 0.002	−*0.921*
*Default mode – Limbic*				
L – precuneus to L – amygdala	−0.02 ± 0.17	0.14 ± 0.15	*t* = 3.58, *p* = 0.001	−*1.010*
L – paracentral lobule to L – amygdala	0.07 ± 0.18	0.21 ± 0.13	*t* = 3.52, *p* = 0.001	−*0.979*
*Somatomotor – Cingulo-frontoparietal*				
L – supplementary motor area to L – superior frontal gyrus	0.26 ± 0.17	0.43 ± 0.20	*t* = 3.25, *p* = 0.002	−*0.916*
L – supplementary motor area to L – superior orbitofrontal gyrus	0.02 ± 0.21	0.20 ± 0.20	*t* = 3.08, *p* = 0.003	−*0.862*
L – supplementary motor area to R – superior orbitofrontal gyrus	−0.03 ± 0.17	0.14 ± 0.19	*t* = 3.38, *p* = 0.001	−*0.938*
R – supplementary motor area to R – superior orbitofrontal gyrus	0.01 ± 0.15	0.18 ± 0.16	*t* = 3.73, *p* < 0.001	−*1.052*
*Somatomotor – Ventral attention*				
R – supplementary motor area to R – inferior orbitofrontal gyrus	0.19 ± 0.20	0.38 ± 0.19	*t* = 3.54, *p* = 0.001	−*0.999*
R – supplementary motor area to R – inferior frontal triangularis	0.11 ± 0.19	0.29 ± 0.20	*t* = 3.37, *p* = 0.001	−*0.936*
L – supplementary motor area to R – inferior orbitofrontal gyrus	0.23 ± 0.18	0.42 ± 0.18	*t* = 3.75, *p* < 0.001	−*1.045*
*Somatomotor – Dorsal attention*				
L – supplementary motor area to L – middle frontal gyrus	0.16 ± 0.17	0.35 ± 0.20	*t* = 3.52, *p* = 0.001	−*0.976*
R – supplementary motor area to R – middle frontal gyrus	0.11 ± 0.21	0.30 ± 0.18	*t* = 3.50, *p* = 0.001	−*0.986*

*Mean and standard deviations for the connectivity strengths for the different connections defined using the AAL atlas. The significant network connections identified as significant were applied to the significant networks extracted from the functional parcelation template based on AAL and MNI coordinates.*

#### Within Network Connectivity Differences of the Default Mode Network Between the Combined and Inattentive Attention Deficit Hyperactivity Disorder Types

**FIGURE 2:**
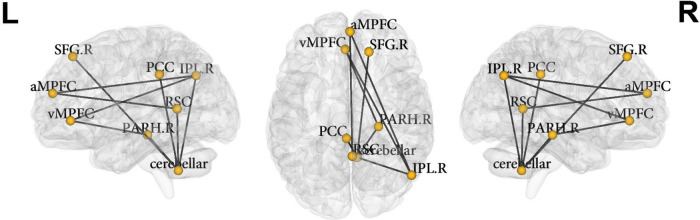
Nodes of the default mode network identified to be significantly different between ADHD-C and ADHD-I groups using NBS. vMPFC, ventral medial prefrontal cortex; aMPFC, anterior medial prefrontal cortex; PCC, posterior cingulate cortex; IPL.R, inferior lateral parietal (right); SFG.R, superior frontal gyrus (right); PARH.R, parahippocampus (right); cerebellar, cerebellar tonsils; RSC, retrosplenial cortex; L, left; R, right.

Network-based statistical analysis identified a connectomic signature comprising 9 connections across 8 nodes between ADHD-C and ADHD-I types (ADHD-C < ADHD-I; Figure 2 and Table 3; *p* = 0.031, corrected for multiple comparisons) within the DMN characterized by: reduced intra-network intrinsic functional connectivity for the ADHD-C group for anterior-posterior DMN regions and between the cerebellar tonsils and para-hippocampus, and between the cerebellar tonsils and superior frontal and posterior cingulate cortices. No within-network differences were found between either ADHD type compared with controls.

**TABLE 3:** Nodes of the default mode network identified to be significantly different between ADHD-C and ADHD-I groups using network-based statistical analysis.

Significant network connections (AAL Regions)	Intrinsic functional connectivity			
	ADHD-C (*n* = 19)	ADHD-I (*n* = 15)		
	Mean ± SD	Mean ± SD	*t* and *p*-value	*Cohen’s d*
Ventral mPFC and R – lateral parietal	0.04 ± 0.19	0.18 ± 0.14	*t* = 2.28, *p* = 0.030	−0.782
Anterior mPFC and R – lateral parietal	0.22 ± 0.27	0.42 ± 0.25	*t* = 2.25, *p* = 0.030	−0.783
Ventral mPFC and R – parahippocampus	0.08 ± 0.23	0.23 ± 0.17	*t* = 2.19, *p* = 0.034	−0.764
R – parahippocampus and cerebellar tonsils	−0.05 ± 0.15	0.17 ± 0.15	*t* = 2.24, *p* = 0.030	−0.975
R – lateral parietal cortex and cerebellar tonsils	−0.09 ± 0.14	0.04 ± 0.11	*t* = 3.04, *p* = 0.005	−1.047
R – superior frontal cortex and cerebellar tonsils	−0.06 ± 0.14	0.05 ± 0.14	*t* = 2.13, *p* = 0.039	−0.744
Anterior mPFC and retrosplenial	0.36 ± 0.17	0.50 ± 0.22	*t* = 2.04, *p* = 0.051	−0.700
Cerebellar tonsils and retrosplenial	−0.05 ± 0.20	0.08 ± 0.18	*t* = 2.00, *p* = 0.052	−0.697
Posterior cingulate cortex and cerebellar tonsils	−0.16 ± 0.20	0.01 ± 0.12	*t* = 2.80, *p* = 0.006	−0.975

*Mean and standard deviations for the connectivity strengths of the significant different connections in the default mode network, ventral mPFC; ventral medial prefrontal cortex, anterior mPFC; anterior medial prefrontal cortex, L, left; R, right.*

### Graph Theoretical Analyses

Clustering coefficient and global characteristic path length did not differ between ADHD types or either of the ADHD types and controls (all *q* > 0.05). There were no significant differences in regional nodal degree between ADHD types or controls after correcting for multiple comparisons (all *q* > 0.05). Differences at an uncorrected threshold comparing the two ADHD types (Supplementary Figure 1A), ADHD-C compared to controls (Supplementary Figure 1B), and ADHD-I compared to controls (Supplementary Figure 1C) have been illustrated in the Supplementary Material. Similarly, the global graph network properties of the DMN did not significantly differ between all three groups.

### Correlations Between Significant Connectivity Measures With ADHD-RS Symptom Severity Scores

#### Whole Brain Network-Based Analysis and ADHD-RS Scores in ADHD-C

We performed a correlation analysis using the significant network edges revealed in the whole brain NBS analysis between the ADHD-C type and controls and the ADHD-RS clinical measures. None of the network connections significantly correlated with ADHD symptom severity (all *q* > 0.05); associations identified at the uncorrected level are summarized in Supplementary Table 1.

#### Default Mode Network Connectivity and ADHD-RS Correlations

Default mode network connections identified from the NBS analysis as significantly different between ADHD-C and ADHD-I were correlated with ADHD-RS clinical rating measures. Reduced functional connectivity of the DMN was associated with higher ADHD-RS hyperactive-impulsive subscale score (*q* = 0.040; Table 4) specifically with reduced connectivity for anterior-posterior DMN regions (ventral medial prefrontal cortex and lateral parietal cortices; ventral medial prefrontal cortex and para-hippocampus); and within posterior DMN regions (para-hippocampus and cerebellar tonsils; lateral parietal cortices, and cerebellar tonsils). There were no correlations between DMN nodal connectivity and ADHD-RS Inattentive symptom severity or ADHD-RS total item scores.

**TABLE 4:** Correlations between connectivity for the links of the default mode network identified to be significantly different between the ADHD-C and ADHD-I Groups and the ADHD-RS scores.

ADHD combined and predominantly inattentive type participants (*n* = 34)									
	***Inattentive subscale score***			***Hyp-imp subscales***			***Total item score***		
	*r* ^2^	*p*	*q*	*r* ^2^	*p*	*q*	*r* ^2^	*p*	*q*
Ventral mPFC and R – lateral parietal	−0.02	0.899	0.976	−0.45	0.010*	0.040**	−0.43	0.015*	0.110
Anterior mPFC and R – lateral parietal	0.01	0.941	0.976	−0.23	0.203	0.203	−0.21	0.255	0.283
Ventral mPFC and R – parahippocampus	0.10	0.570	0.976	−0.46	0.008*	0.040**	−0.37	0.034*	0.113
Posterior cingulate cortex and cerebellar tonsils	−0.11	0.539	0.976	−0.28	0.126	0.158	−0.31	0.082	0.164
R – lateral parietal cortex and cerebellar tonsils	0.01	0.976	0.976	−0.44	0.012*	0.040**	−0.40	0.022*	0.110
R – superior frontal cortex and cerebellar tonsils	0.02	0.904	0.976	−0.32	0.071	0.135	−0.29	0.109	0.182
R – parahippocampus and cerebellar tonsils	0.31	0.086	0.430	−0.42	0.016*	0.040**	−0.24	0.190	0.238

Significant differences between the ADHD-C and ADHD-I subtypes at p < 0.05 are highlighted in bold. *Association significant at the uncorrected level p < 0.05. **Association significant at the corrected level q < 0.05. ADHD-RS; rating scales, ventral mPFC; ventral medial prefrontal cortex, anterior mPFC; anterior medial prefrontal cortex; ADHD-C, ADHD combined presentation; ADHD-I, ADHD predominantly inattentive presentation; ADHD-RS IV, attention deficit hyperactivity disorder rating scales-version 4; ADHD-RS IV Int Subscale, inattentive subscale score; ADHD-RS IV Hyp-Imp subscales score, hyperactivity-impulsivity subscales score.

## Discussion

We employed a comprehensive, connectome-wide analysis to investigate whether large-scale intrinsic brain functional connectivity networks distinguish the combined (ADHD-C) and inattentive (ADHD-I) presentation types of ADHD using NBS and graph-theoretical metrics. NBS analysis of the whole-brain connectome did not identify significant differences between the ADHD types or the ADHD-I type compared to controls. We identified a functional connectomic signature characterizing ADHD-C participants from controls, with primarily reduced intra-network connectivity within the DMN; and inter-network connectivity between the DMN and somatomotor (SSN) and limbic networks; and somatomotor (SSN) and cingulo-frontoparietal (CFP), ventral, and dorsal attention networks. Within network connectivity analysis of the DMN also revealed connectivity differences between the ADHD-C and ADHD-I groups. However, these connectivity differences were observed in the context of no differences in global or regional network properties between the two ADHD types, utilizing graph-theoretical analysis. Additionally, within network connectivity of the DMN and ADHD-RS hyperactive-impulsive symptom subscale scores were negatively correlated.

Previous resting-state fMRI studies have demonstrated impaired connectivity in large-scale functional brain networks in ADHD, distinguishing ADHD types from each other (Fair et al., 2012; Qian et al., 2019; Zhou et al., 2019; Ahmadi et al., 2021). In addition, studies have reported decreased functional connectivity between cingulo-frontoparietal, ventral attention, somatomotor and DMN (Zhang et al., 2020), particularly between the anterior DMN and somatomotor networks, characterizing ADHD-C from controls (Fair et al., 2010; dos Santos Siqueira et al., 2014; Qian et al., 2019; Zhou et al., 2019). Our analysis supports these previous findings and provides insights on the differential functional intrinsic network phenotypes in our ADHD-C and ADHD-I types cohort, specifically within the DMN. We also observed altered connectivity related to the DMN, somatomotor and cingulo-frontoparietal networks for the ADHD-C group relative to controls. These findings have implications for a brain network-based framework informing the underlying neurobiological profiles of the ADHD-C and ADHD-I types.

The present study found decreased inter-network connectivity related to the somatomotor network, CFP and DMN brain networks in ADHD-C relative to controls. The regulation of executive function, attention and response inhibition, which clinically characterize ADHD types (Faraone et al., 2015), is proposed to underlie inter-regional network organization in these three networks; SSN, DMN and CFP (Menon, 2011). Involved in the modulation of motor activity response to stimulus, disruptions to the SSN, is also known to interact with the CFP and DMN, and has implications for poor regulation of impulsivity, attentional control and inhibition, which are clinical symptoms characteristic of ADHD-C (Fair et al., 2012; Wang et al., 2020). Interestingly, a study comparing adults with ADHD and children with ADHD found reduced connectivity between the somatomotor network to the dorsal attention network in the ADHD children only (Guo et al., 2020), in line with the knowledge of diminished hyperactive-impulsive symptoms observed in adults with ADHD (Tao et al., 2017). The somatomotor and DMN are understood to reach maturational peaks in early childhood and late adolescence, respectively. Therefore, it is likely that delayed maturation of these networks in ADHD underlying the clinical symptoms characteristic of ADHD-C type is accordant with maturational lag models in ADHD (Shaw et al., 2013; Sudre et al., 2018).

While within network connectivity for the DMN distinguished ADHD-C from controls, reduced connectivity in the network also characterized ADHD-C from ADHD-I type. Specifically, we found reduced inter-nodal anterior-posterior connectivity of the DMN and in posterior regions of the DMN. These regions assist regulation of delayed gratification in social decision making and emotional response, planning, and sensorimotor control (Buckner et al., 2008; Broulidakis et al., 2022). Furthermore, anterior-posterior DMN integration and increased connectivity, especially involving the medial PFC and posterior cingulate cortices of the DMN, are susceptible to maturational trajectories of network connectivity (Buckner et al., 2008; Fair et al., 2008; Washington and VanMeter, 2015). Aligned with previous studies, our findings of reduced connectivity in these DMN nodes may reflect delays in developmental trajectory, reflecting the clinical symptoms associated with ADHD-C (Fair et al., 2012; Sripada et al., 2014a,b; Qian et al., 2019).

This finding supports previous studies demonstrating distinct functional network connectivity mostly in the DMN and somatomotor, characterizing ADHD-C from ADHD-I (Fair et al., 2012; dos Santos Siqueira et al., 2014) and also controls (Fair et al., 2012; dos Santos Siqueira et al., 2014; Gao et al., 2019; Zhang et al., 2020). We also observed reduced connectivity for the DMN and limbic regions for ADHD-C relative to controls. Studies employing a similar combinatorial approach of NBS and graph theory analysis, comparatively to controls, have also found reduced connectivity in the visual attention network and between the DMN in ADHD-C participants (Xia et al., 2014), decreased functional inter-network connectivity between the DMN and frontoparietal network in medication naive ADHD children (Tao et al., 2017).

Reduced connectivity between the three networks of the DMN and somatomotor network interacting with the CFP may be concordant with failure to suppress the DMN, effectively compromising the ability of the cognitive control network to activate and apply to attenuation of the task and motivation (Gao et al., 2019). Moreover, the reduced connectivity for the cerebellar regions of the DMN in ADHD-C type is not a surprising finding, as the cerebellar system is central to the modulation of motor skills and regulation of executive functioning (Faraone et al., 2015), which underpin symptoms characteristic of this type (Saad et al., 2020). The absence of significant inter-regional network differences for the ADHD-I type, compared to controls, is consistent with some previous studies reporting no significant differences (Qian et al., 2019; Zhou et al., 2019), though equivocally, hypoconnectivity between the DAN and DMN has been reported, compared to controls (Zhang et al., 2020). Also, compared to previous studies, we found no differences in graph-theoretic measures (Tao et al., 2017); thus, our study’s absence of graph results may be attributed to smaller sample sizes and differences in network construction methods comparatively with previous studies.

The correlation results confirmed the relationship between aberrant functional connectivity within the DMN and ADHD symptoms in the two ADHD types. Items 10–18 on the ADHD-RS correspond to the hyperactive-impulsive DSM symptom criteria. Significant negative associations were found between connectivity within the DMN, linking the ventral mPFC and right lateral parietal gyrus, ventral mPFC and right parahippocampus, and right parahippocampus and cerebellar tonsils, with hyperactive-impulsive subscale scores. Interestingly, these regions underpin decision making, self-regulation, memory and response inhibition, with the cerebellar tonsils known to play a role in visual-motor and perception, characteristic of ADHD-C clinical symptoms and proposed DMN and visual and motor networks implicated particularly to ADHD-C type (Cao et al., 2014; Icer et al., 2019). Similarly, an inverse association of connectivity in frontal regions of the executive control network and hyperactive-impulsive symptoms in ADHD-C has been previously reported (Elton et al., 2014; Francx et al., 2015; Pruim et al., 2019).

### Limitations

This study has several limitations. First, considering the small sample size of our study, the generalizability of results is limited; thus, replication in larger cohorts is warranted to deduce whether large-scale functional networks differ between the ADHD types, especially at the whole connectome level. We have provided uncorrected findings in the Supplementary Material, as the absence of significant findings from graph theory measures in our study could be likely due to the small available sample size. Data for the predominantly hyperactive-impulsive type was unavailable in our study; therefore, analysis was limited to the combined and inattentive type. Notably, the availability of research on the ADHD-HI type in the literature is significantly less than the ADHD-C type population. In addition, studies explicitly comparing the subtypes are often fewer in number and underpowered, limiting opportunities to establish and replicate findings specifically to the ADHD-HI type. Thus, future research may prioritize the recruitment of the ADHD-HI type in its cohort to explore the shared core hyperactive-impulsive differences across the ADHD-C and ADHD-HI types. Establishing a brain network framework for ADHD types necessitates future neuroimaging research to incorporate cohorts with equitable distribution of the ADHD types. Also, the impact of neurodevelopment on the formation of neural mechanisms and the trajectory of symptoms underlying the two subtypes is not yet well understood and warrants future work. Attentional issues in the ADHD-I type appear somewhat later than the ADHD-C type, while hyperactive-impulsive symptoms diminish with increased age (Biederman et al., 2000; Saad et al., 2020). While this study matched participants for age and gender, the small sample size restricts analyses by developmental periods to explore the possible maturational influence on the network differences. Thus, longitudinal studies examining neurobiological differences based on maturational periods would be informative to understand trajectories of functional brain networks in the ADHD types (Qian et al., 2019; Saad et al., 2020).

Additionally, medication effects and stimulant treatment history may bias our results despite participants undergoing a washout phase before study commencement. It is challenging to eliminate previous treatment effects, which reportedly “normalize” brain maturation by reducing atypical higher resting-state functional network connectivity (Silk et al., 2017). Thus, while treatment experience may minimize significant differences in our study, the extent of medication effects on functional connectivity remains inconclusive (Albajara Sáenz et al., 2019).

## Conclusion

In summation, our study used intrinsic functional connectomic analyses incorporating network and graph-theoretic analyses underlying the two most common clinical ADHD presentation types, ADHD-C and ADHD-I. The new insights this study provides into functional intrinsic network phenotypic differences between the two types revealed within network differences in the DMN; and large scale brain inter-regional network differences between the somatomotor cingulo-frontoparietal and default mode networks, in between ADHD-Combined and controls. Thus, contributing to the burgeoning evidence of large-scale brain network topology in ADHD types, our findings potentiate distinct neurobiological features underlying the ADHD types in line with the literature. Furthermore, understanding the neural network architecture underlying the ADHD types provides evidence of differential network connectivity between the ADHD symptom presentations. This has significant implications for developing differential treatments suited for one type versus another.

## Data Availability Statement

The original contributions presented in this study are included in the article/Supplementary Material, further inquiries can be directed to the corresponding author/s.

## Ethics Statement

The studies involving human participants were reviewed and approved by the Western Sydney Local Health District Human Research Ethics Committee. Written informed consent to participate in this study was provided by the participants’ legal guardian/next of kin.

## Author Contributions

MSK, LW, MRK, SC, and KG contributed to the participant recruitment, clinical assessment, MRI data collection, and processing and management. JFS performed the data analysis and interpretation of results, with contributions from TB and MSK, and contributed to preparing and writing the original manuscript draft. JFS, MSK, LW, KG, and TB contributed to the review and editing of the manuscript. All authors contributed to the article and approved the submitted version.

## Conflict of Interest

JFS, KG, MRK, and SC have received honoraria for educational seminars from Takeda (Shire – Australia). LW has received consultant fees from BlackThorn Therapeutics and scientific advisory board fees from Psyberguide of the One Mind Institute. MSK, KG, MRK, and SC have received commercial funding from Takeda for funded project grants. The remaining author declare that the research was conducted in the absence of any commercial or financial relationships that could be construed as a potential conflict of interest.

## Publisher’s Note

All claims expressed in this article are solely those of the authors and do not necessarily represent those of their affiliated organizations, or those of the publisher, the editors and the reviewers. Any product that may be evaluated in this article, or claim that may be made by its manufacturer, is not guaranteed or endorsed by the publisher.

## Footnotes

1
http://www.fil.ion.ucl.ac.uk/spm/ext/#TSDiffAna

2
http://www.brain-connectivity-toolbox.net/

3
http://www.nitrc.org/projects/bnv/
