# Supplementary material for: Intrinsic Functional Connectivity in the Default Mode Network Differentiates the Combined and Inattentive Attention Deficit Hyperactivity Disorder Types
Source: Front Hum Neurosci. 2022 Jun 9;16:859538. doi: 10.3389/fnhum.2022.859538 (PMC9218495; doi:10.3389/fnhum.2022.859538)
Supplement: Supplementary file 1 [file Data_Sheet_1.docx]

Figure 1

**Figure S1A.** **The regional nodal degree between ADHD types or relative to controls after correcting for multiple comparisons (all q > .05). Differences at an uncorrected threshold comparing the two ADHD types.**


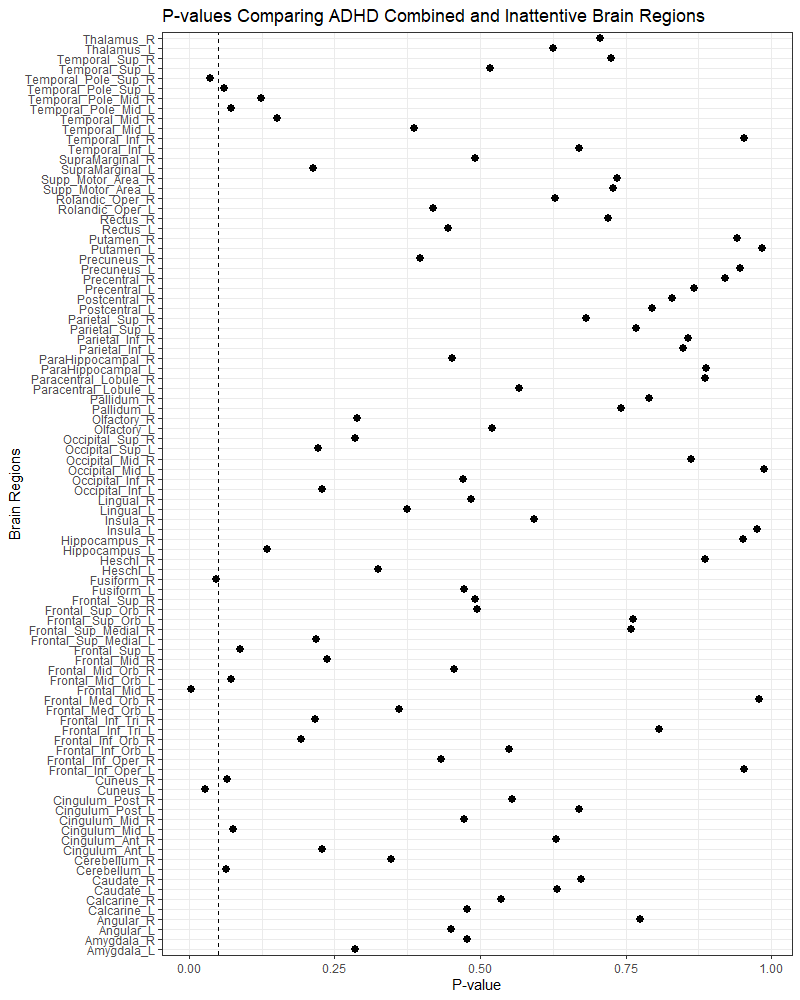


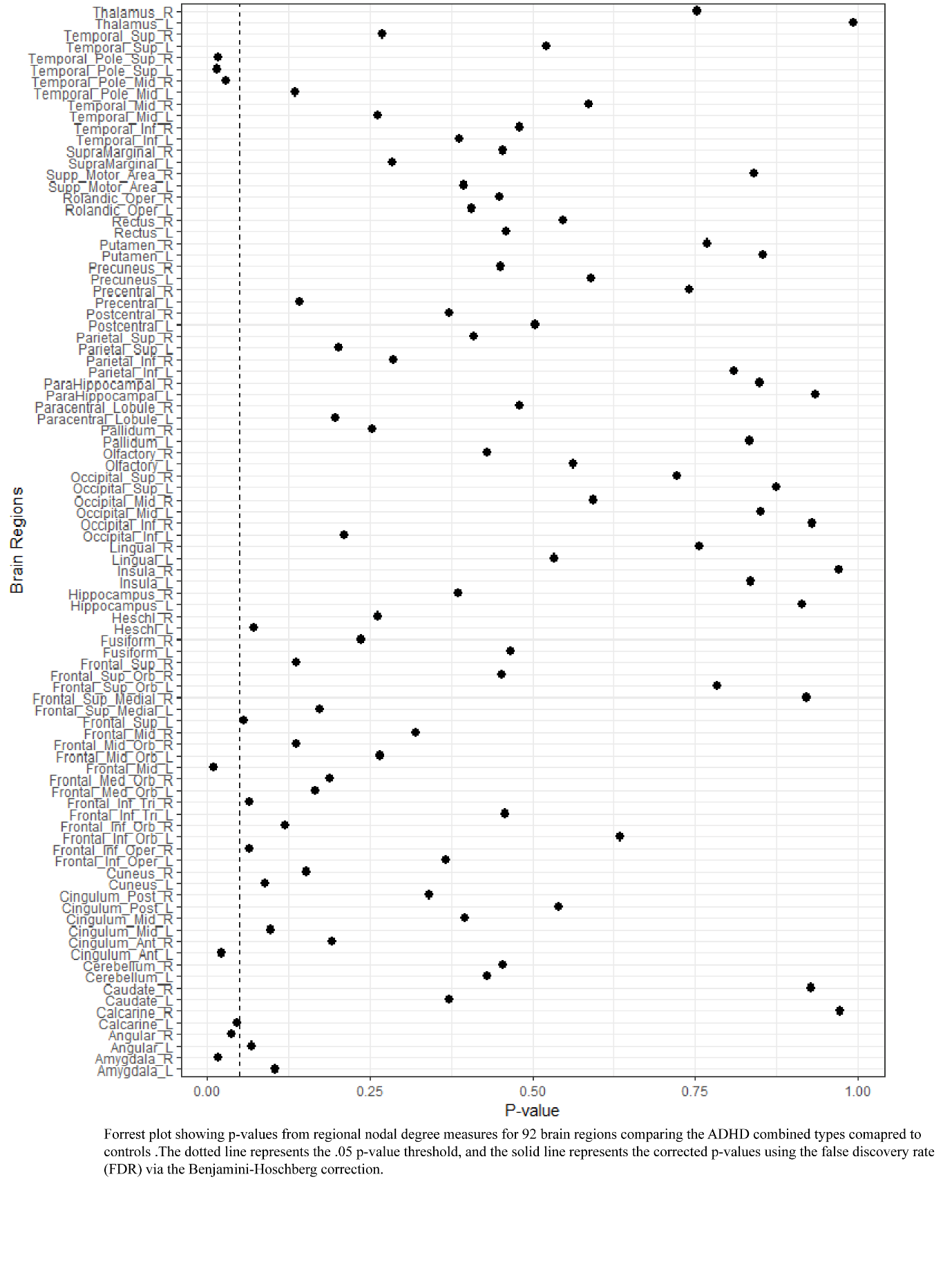
**Figure S1B.** **The regional nodal degree between ADHD-C relative to controls after correcting for multiple comparisons (all q > .05). Differences at an uncorrected threshold comparing ADHD-C and controls.**

**Figure S1C. The regional nodal degree between ADHD-I relative to controls after correcting for multiple comparisons (all q > .05). Differences at an uncorrected threshold comparing ADHD-I and controls.**


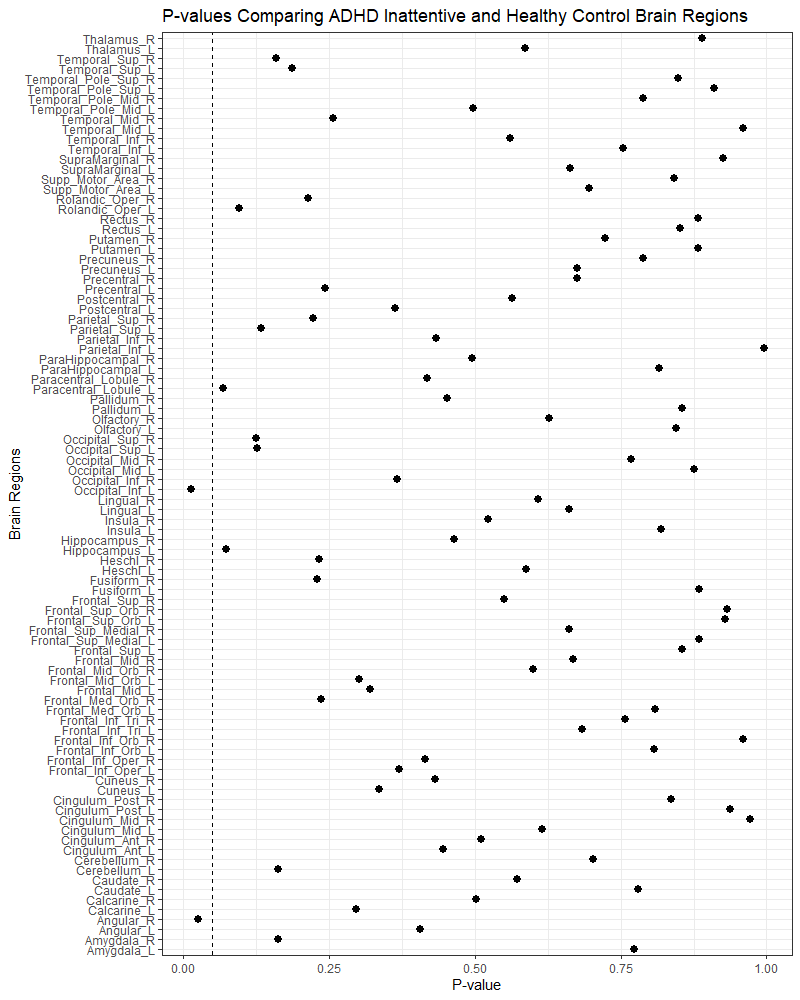


Figure s2.

Distribution of a single significant DMN connectivity score between the ADHD-C and ADHD-I types based on NBS analysis.


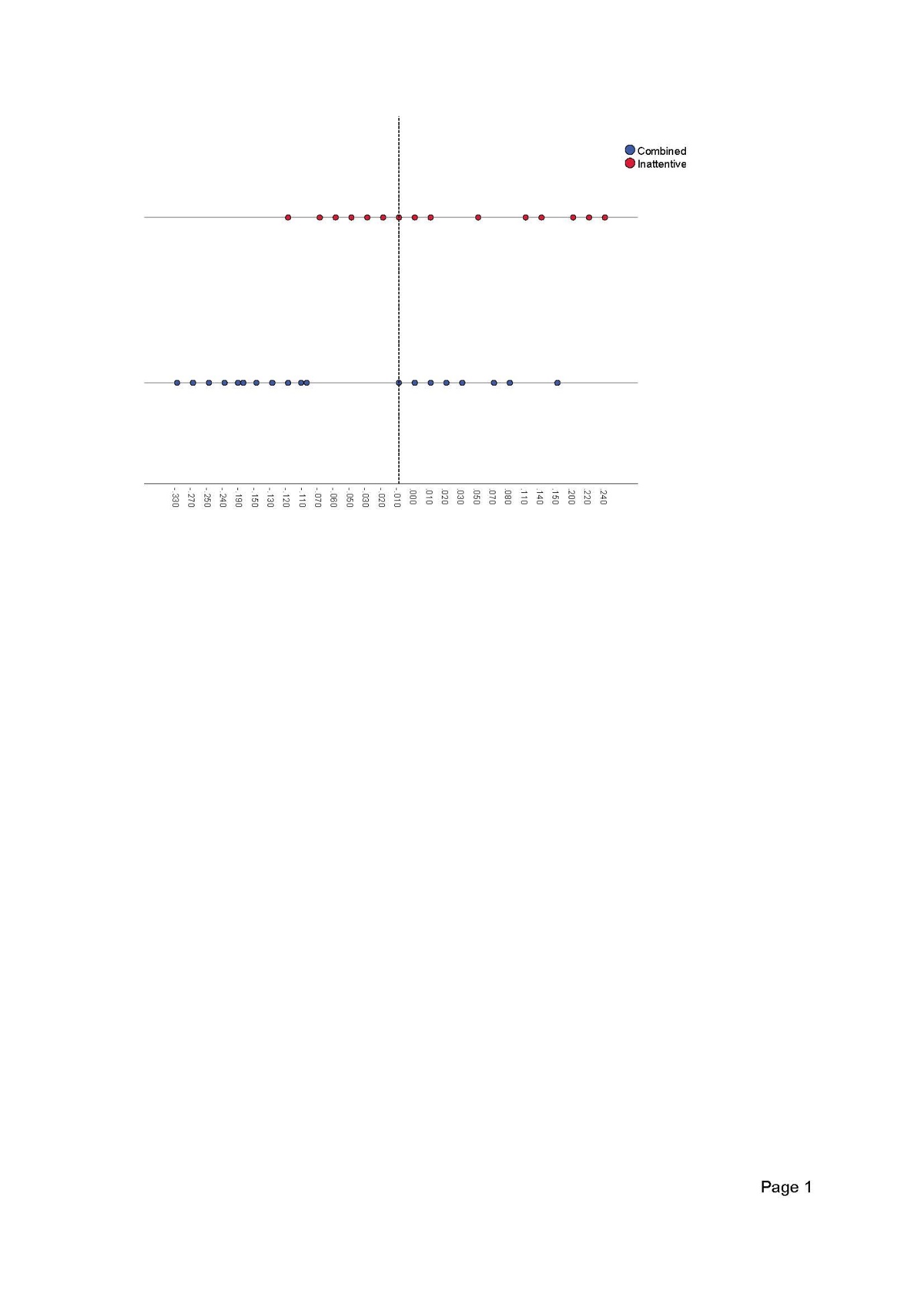


# Figures S3. Scatterplots of adhd-rs iv sum of inattentive scores, sum of hyperactive-impulsive scores, and total scores

A. ADHD-RS -IV Sum Of Items 1 To 9: Inattention Scores


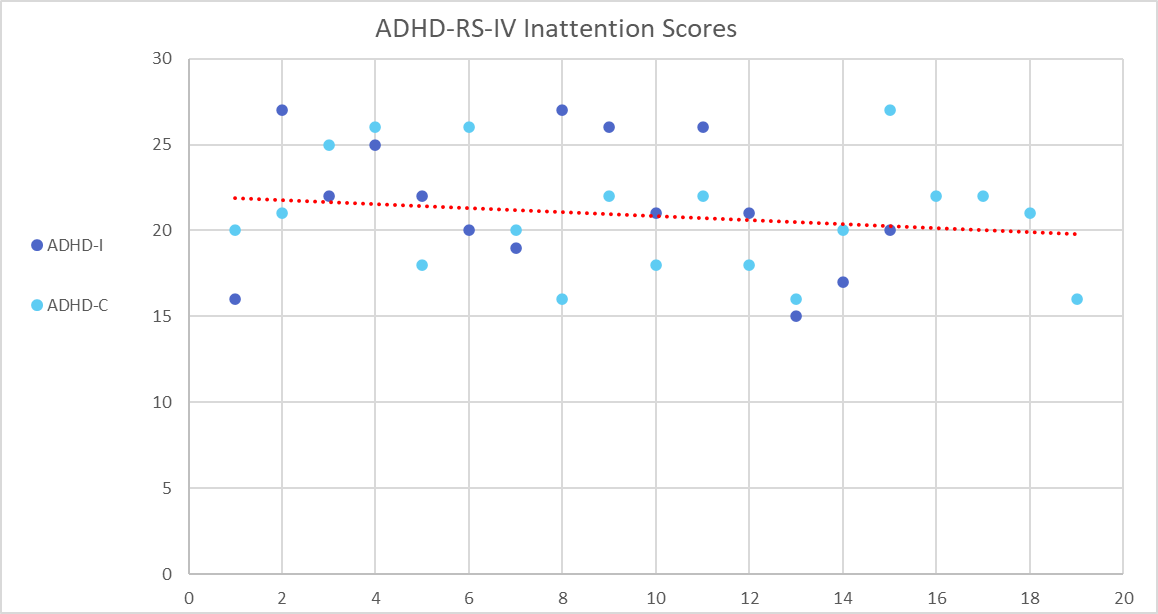


B. ADHD-RS -IV Sum Of Items 10 to 18: Hyperactive-Impulsive Scores


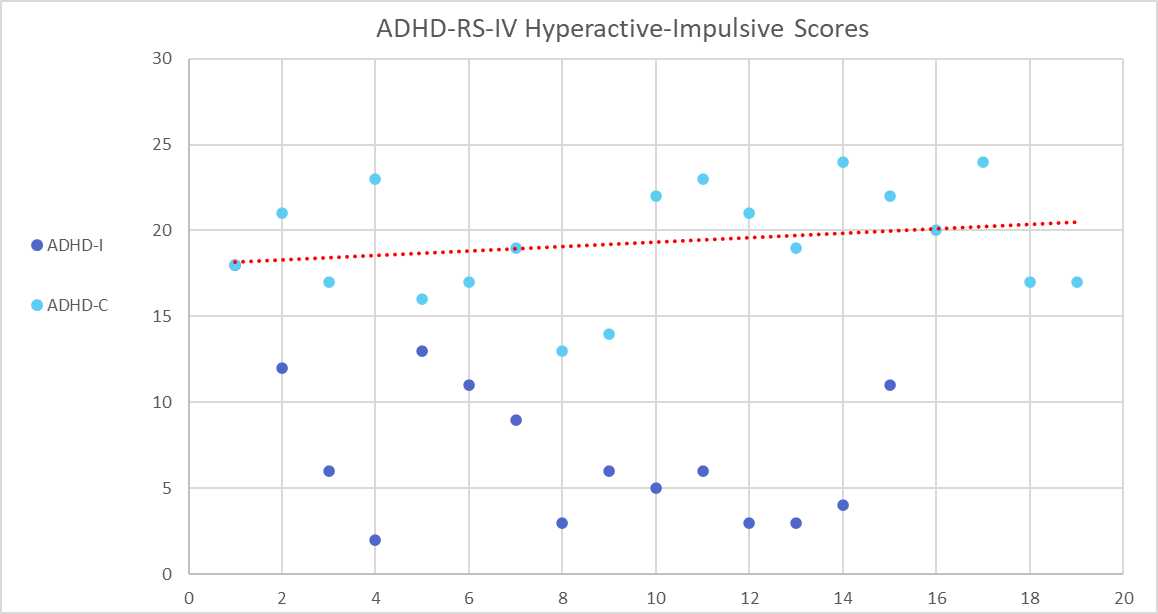


c. ADHD-RS-IV Distribution of Inattention and Hyperactive-Impulsive Scores


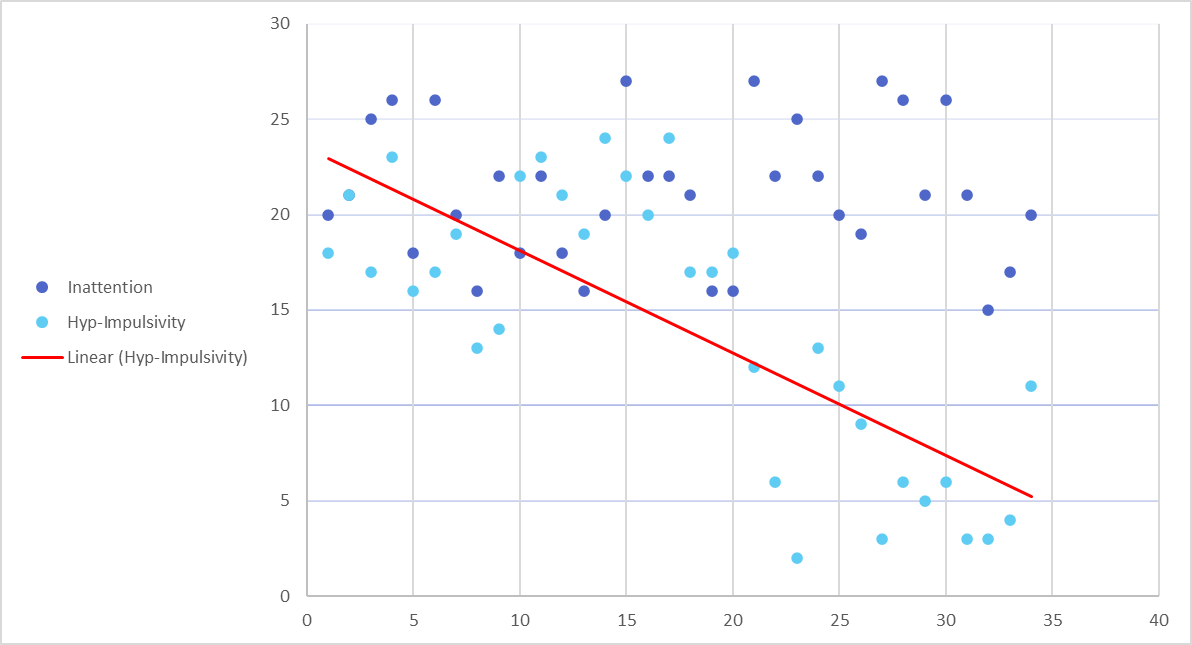


Correlations between the ADHD-RS IV scores and global whole-brain and regional nodal degree graph measures, and NBS whole brain and DMN significant networks

S1 Table. Correlations between the whole-brain networks identified to be significantly different and the ADHD-RS IV scores between the ADHD Combined and Control groups using Network-Based Statistical Analysis.

| **ADHD Combined type (*n* = 19) and Controls (*n* = 39)** | | | | | | | | | | |
| --- | --- | --- | --- | --- | --- | --- | --- | --- | --- | --- |
|  | ADHD-RS Sum of Items 1 - 9 | | | ADHD- RS Sum of Items 10 - 18 | | | Total Item Score | | | |
|  | *r^2^* | *p* | *q* | *r^2^* | *p* | *q* | | *r^2^* | *p* | *q* |
| R-Frontal_Inf_Orb and L-Supp_Motor_Area | .34 | .176 | .578 | .21 | .427 | .844 | | .35 | .174 | .906 |
| L-Frontal_Mid and L-Supp_Motor_Area | .30 | .235 | .578 | -.25 | .333 | .844 | | .05 | .852 | .956 |
| L-Frontal_Sup and L-Supp_Motor_Area | .41 | .104 | .564 | -.20 | .448 | .844 | | .15 | .572 | .950 |
| L-Frontal_Sup_Medial and L-Supp_Motor_Area | .51 | .035* | .564 | .06 | .810 | .926 | | .37 | .144 | .906 |
| R-Frontal_Sup_Medial and L-Supp_Motor_Area | .37 | .141 | .564 | .04 | .873 | .952 | | .27 | .302 | .906 |
| L-Frontal_Sup_Orb and L-Supp_Motor_Area | .14 | .603 | .899 | -.19 | .457 | .844 | | -.03 | .919 | .956 |
| R-Frontal_Sup_Orb and L-Supp_Motor_Area | -.05 | .862 | .899 | -.35 | .164 | .844 | | -.24 | .356 | .943 |
| R-Frontal_Inf_Orb and R-Supp_Motor_Area | .30 | .241 | .578 | .17 | .519 | .863 | | .29 | .251 | .906 |
| R-Frontal_Inf_Tri and R-Supp_Motor_Area | .14 | .596 | .899 | .52 | .032* | .768 | | .40 | .113 | .906 |
| R-Frontal_Mid_Orb and R-Supp_Motor_Area | .33 | .202 | .578 | .11 | .686 | .863 | | .27 | .287 | .906 |
| R-Frontal_Mid and R-Supp_Motor_Area | .03 | .901 | .901 | -.11 | .664 | .863 | | -.05 | .860 | .956 |
| R-Frontal_Sup_Medial and R-Supp_Motor_Area | .08 | .751 | .899 | -.38 | .132 | .844 | | -.17 | .511 | .943 |
| R-Frontal_Sup_Orb and R-Supp_Motor_Area | .40 | .113 | .564 | -.09 | .719 | .863 | | .20 | .434 | .943 |
| L-Precentral and L-Cingulum_Ant | .25 | .334 | .688 | .26 | .313 | .844 | | .32 | .216 | .906 |
| L-Supp_Motor_Area and L-Cingulum_Ant | .07 | .775 | .899 | -.27 | .298 | .844 | | -.11 | .673 | .950 |
| R-Frontal_Mid_Orb and R-Temporal_Inf | -.11 | .665 | .899 | -.24 | .354 | .844 | | -.22 | .406 | .943 |
| L-Supp_Motor_Area and R-Temporal_Inf | .08 | .770 | .899 | .02 | .949 | .961 | | .06 | .820 | .956 |
| R-Supp_Motor_Area and R-Temporal_Inf | .24 | .344 | .688 | -.23 | .379 | .844 | | .02 | .928 | .956 |
| R-Frontal_Mid_Orb and R-Temporal_Mid | -.07 | .787 | .899 | .10 | .697 | .863 | | .01 | .956 | .956 |
| L-Cingulum_Ant and R-Paracentral Lobule | .19 | .472 | .871 | -.13 | .621 | .863 | | .04 | .865 | .956 |
| L-Amygdala and L-Paracentral_Lobule | -.17 | .522 | .895 | -.01 | .961 | .961 | | -.12 | .658 | .950 |
| L-Cingulum_Ant and L-Rectus | .05 | .851 | .899 | .40 | .111 | .844 | | .27 | .296 | .906 |
| L-Amygdala and L-Precuneus | -.38 | .138 | .564 | .21 | .421 | .844 | | -.12 | .648 | .950 |
| L-Rectus and R-Cerebellum_combined | .40 | .116 | .564 | -.14 | .592 | .863 | | .17 | .505 | .943 |

*association significant at the uncorrected level p < .05

** association significant at the FDR corrected level q < .05

S2 Table. Correlations between the global network measures and the ADHD RS-IV item scores of children and adolescents with ADHD combined and ADHD inattentive types.

| **ADHD Combined and ADHD Predominately Inattentive Type Participants (*n* = 34)** | | | |
| --- | --- | --- | --- |
|  | *r^2^* | *p* | *q* |
| **ADHD-RS Sum of Items 1 - 9** |  |  |  |
| AUC_ Mean Clustering Coefficient | .34 | .057 | .674 |
| AUC_Characteristic Path Length | .37 | .039 | .556 |
| **ADHD- RS Sum of Items 10 - 18** |  |  |  |
| AUC_ Mean Clustering coefficient | - .12 | .508 | .856 |
| AUC_ Characteristic path length | -.1 | .586 | .856 |
| **Total Item Score** |  |  |  |
| AUC_ Mean Clustering coefficient | .06 | .755 | .851 |
| AUC_Characteristic path length | .09 | .623 | .797 |

AUC, area under the curve; ADHD-RS IV, attention deficit hyperactivity disorder rating scales - version 4; ADHD-RS Sum of Items 1 -9, inattention subscale; ADHD- RS Sum of Items 10 – 18, hyperactivity-impulsivity subscale; Total item score, the sum of 18 items involving the inattention and hyperactivity-impulsivity subscale.

S3 Table. Correlations between the Regional Nodal Degree measures for 92 brain regions and the ADHD-RS IV Inattentive subscale scores of children and adolescents with ADHD combined and ADHD inattentive types.

| ADHD combined and ADHD inattentive type participants (*n* = 34) | | | | | |
| --- | --- | --- | --- | --- | --- |
|  | *r^2^* | | | *p* | *q* |
| Amygdala_L | | -.37 | .039* | | .556 |
| Amygdala_R | | -.21 | .260 | | .842 |
| Angular_L | | -.08 | .656 | | .875 |
| Angular_R | | -.12 | .524 | | .844 |
| Calcarine_L | | -.38 | .030* | | .556 |
| Calcarine_R | | -.22 | .233 | | .842 |
| Caudate_L | | -.13 | .483 | | .842 |
| Caudate_R | | -.13 | .466 | | .842 |
| Cerebellum_L | | -.17 | .339 | | .842 |
| Cerebellum_R | | .09 | .638 | | .866 |
| Cingulum_Ant_L | | -.07 | .692 | | .888 |
| Cingulum_Ant_R | | -.09 | .631 | | .866 |
| Cingulum_Mid_L | | -.24 | .187 | | .842 |
| Cingulum_Mid_R | | -.17 | .344 | | .842 |
| Cingulum_Post_L | | -.12 | .503 | | .842 |
| Cingulum_Post_R | | -.21 | .253 | | .842 |
| Cuneus_L | | -.19 | .288 | | .842 |
| Cuneus_R | | -.03 | .856 | | .927 |
| Frontal_Inf_Oper_L | | -.07 | .711 | | .901 |
| Frontal_Inf_Oper_R | | -.09 | .617 | | .866 |
| Frontal_Inf_Orb_L | | -.14 | .432 | | .842 |
| Frontal_Inf_Orb_R | | .01 | .959 | | .959 |
| Frontal_Inf_Tri_L | | .12 | .497 | | .842 |
| Frontal_Inf_Tri_R | | .14 | .455 | | .842 |
| Frontal_Med_Orb_L | | .17 | .356 | | .842 |
| Frontal_Med_Orb_R | | .27 | .142 | | .842 |
| Frontal_Mid_L | | .06 | .751 | | .915 |
| Frontal_Mid_Orb_L | | .04 | .811 | | .927 |
| Frontal_Mid_Orb_R | | .17 | .354 | | .842 |
| Frontal_Mid_R | | .13 | .475 | | .842 |
| Frontal_Sup_L | | -.03 | .868 | | .927 |
| Frontal_Sup_Medial_L | | -.04 | .821 | | .927 |
| Frontal_Sup_Medial_R | | -.40 | .025* | | .556 |
| Frontal_Sup_Orb_L | | -.19 | .295 | | .842 |
| Frontal_Sup_Orb_R | | .16 | .369 | | .842 |
| Frontal_Sup_R | | .13 | .467 | | .842 |
| Fusiform_L | | -.17 | .356 | | .842 |
| Fusiform_R | | -.08 | .666 | | .875 |
| Heschl_L | | .22 | .232 | | .842 |
| Heschl_R | | -.04 | .843 | | .927 |
| Hippocampus_L | | .05 | .796 | | .927 |
| Hippocampus_R | | -.05 | .779 | | .927 |
| Insula_R | | .31 | .086 | | .681 |
| Insula_L | | -.02 | .919 | | .959 |
| Lingual_L | | -.15 | .413 | | .842 |
| Lingual_R | | .09 | .619 | | .866 |
| Occipital_Inf_L | | -.16 | .381 | | .842 |
| Occipital_Inf_R | | -.21 | .250 | | .842 |
| Occipital_Mid_L | | .01 | .959 | | .959 |
| Occipital_Mid_R | | -.16 | .378 | | .842 |
| Occipital_Sup_L | | -.08 | .672 | | .875 |
| Occipital_Sup_R | | -.14 | .456 | | .842 |
| Olfactory_L | | -.22 | .231 | | .842 |
| Olfactory_R | | .05 | .784 | | .927 |
| Pallidum_L | | -.26 | .145 | | .842 |
| Pallidum_R | | -.13 | .467 | | .842 |
| Paracentral_Lobule_L | | .25 | .175 | | .842 |
| Paracentral_Lobule_R | | .20 | .266 | | .842 |
| ParaHippocampal_L | | -.10 | .580 | | .866 |
| ParaHippocampal_R | | -.03 | .867 | | .927 |
| Parietal_Inf_L | | -.31 | .086 | | .681 |
| Parietal_Inf_R | | -.10 | .591 | | .866 |
| Parietal_Sup_L | | .01 | .936 | | .959 |
| Parietal_Sup_R | | -.04 | .822 | | .927 |
| Postcentral_L | | .09 | .632 | | .866 |
| Postcentral_R | | .22 | .233 | | .842 |
| Precentral_L | | -.13 | .495 | | .842 |
| Precentral_R | | .01 | .937 | | .959 |
| Precuneus_L | | .11 | .557 | | .866 |
| Precuneus_R | | .13 | .480 | | .842 |
| Putamen_L | | .36 | .041* | | .556 |
| Putamen_R | | .23 | .201 | | .842 |
| Rectus_L | | .16 | .394 | | .842 |
| Rectus_R | | .18 | .321 | | .842 |
| Rolandic_Oper_L | | .32 | .071 | | .674 |
| Rolandic_Oper_R | | .33 | .064 | | .674 |
| Supp_Motor_Area_L | | .03 | .854 | | .927 |
| Supp_Motor_Area_R | | .12 | .508 | | .842 |
| SupraMarginal_L | | .12 | .510 | | .842 |
| SupraMarginal_R | | .06 | .734 | | .906 |
| Temporal_Inf_L | | .10 | .601 | | .866 |
| Temporal_Inf_R | | .06 | .729 | | .906 |
| Temporal_Mid_L | | .41 | .018* | | .556 |
| Temporal_Mid_R | | .13 | .483 | | .842 |
| Temporal_Pole_Mid_L | | .11 | .561 | | .866 |
| Temporal_Pole_Mid_R | | -.22 | .222 | | .842 |
| Temporal_Pole_Sup_L | | -.21 | .249 | | .842 |
| Temporal_Pole_Sup_R | | -.15 | .408 | | .842 |
| Temporal_Sup_L | | .42 | .017* | | .556 |
| Temporal_Sup_R | | .14 | .452 | | .842 |
| Thalamus_L | | .10 | .590 | | .866 |
| Thalamus_R | | .12 | .514 | | .842 |
|  | |  |  | |  |

*association significant at the uncorrected level p < .05

** association significant at the FDR corrected level q < .05

S4 Table. Correlations between the Regional Nodal Degree measures for 92 brain regions and the ADHD-RS IV Hyperactive-Impulsive subscale scores of children and adolescents with ADHD combined and ADHD inattentive types.

| ADHD combined and ADHD inattentive type participants (n = 34) | | | |  | |  |  |
| --- | --- | --- | --- | --- | --- | --- | --- |
|  | *r^2^* | | | | *p* | | *q* |
| Amygdala_L | | .19 | .302 | | | | .765 |
| Amygdala_R | | .21 | .257 | | | | .765 |
| Angular_L | | -.11 | .539 | | | | .856 |
| Angular_R | | .06 | .761 | | | | .924 |
| Calcarine_L | | .08 | .670 | | | | .898 |
| Calcarine_R | | .16 | .367 | | | | .792 |
| Caudate_L | | -.18 | .312 | | | | .765 |
| Caudate_R | | -.22 | .226 | | | | .765 |
| Cerebellum_L | | -.29 | .111 | | | | .747 |
| Cerebellum_R | | -.06 | .729 | | | | .918 |
| Cingulum_Ant_L | | -.11 | .546 | | | | .856 |
| Cingulum_Ant_R | | -.13 | .465 | | | | .856 |
| Cingulum_Mid_L | | -.28 | .118 | | | | .747 |
| Cingulum_Mid_R | | -.20 | .262 | | | | .765 |
| Cingulum_Post_L | | -.26 | .156 | | | | .765 |
| Cingulum_Post_R | | -.10 | .572 | | | | .856 |
| Cuneus_L | | -.39 | .027* | | | | .644 |
| Cuneus_R | | -.35 | .051 | | | | .644 |
| Frontal_Inf_Oper_L | | -.17 | .338 | | | | .765 |
| Frontal_Inf_Oper_R | | -.20 | .275 | | | | .765 |
| Frontal_Inf_Orb_L | | .29 | .103 | | | | .747 |
| Frontal_Inf_Orb_R | | .34 | .056 | | | | .644 |
| Frontal_Inf_Tri_L | | .20 | .280 | | | | .765 |
| Frontal_Inf_Tri_R | | .37 | .036* | | | | .644 |
| Frontal_Med_Orb_L | | .01 | .946 | | | | .977 |
| Frontal_Med_Orb_R | | .22 | .233 | | | | .765 |
| Frontal_Mid_L | | -.46 | .008* | | | | .644 |
| Frontal_Mid_Orb_L | | -.33 | .061 | | | | .644 |
| Frontal_Mid_Orb_R | | -.15 | .405 | | | | .819 |
| Frontal_Mid_R | | -.16 | .391 | | | | .807 |
| Frontal_Sup_L | | -.40 | .023* | | | | .644 |
| Frontal_Sup_Medial_L | | -.35 | .049 | | | | .644 |
| Frontal_Sup_Medial_R | | -.05 | .768 | | | | .924 |
| Frontal_Sup_Orb_L | | -.01 | .974 | | | | .991 |
| Frontal_Sup_Orb_R | | .14 | .448 | | | | .851 |
| Frontal_Sup_R | | .19 | .305 | | | | .765 |
| Fusiform_L | | -.27 | .143 | | | | .765 |
| Fusiform_R | | .24 | .188 | | | | .765 |
| Heschl_L | | .10 | .583 | | | | .856 |
| Heschl_R | | -.09 | .642 | | | | .897 |
| Hippocampus_L | | .14 | .437 | | | | .851 |
| Hippocampus_R | | -.06 | .741 | | | | .918 |
| Insula_R | | .08 | .671 | | | | .898 |
| Insula_L | | -.03 | .890 | | | | .970 |
| Lingual_L | | -.20 | .273 | | | | .765 |
| Lingual_R | | -.25 | .162 | | | | .765 |
| Occipital_Inf_L | | .21 | .246 | | | | .765 |
| Occipital_Inf_R | | -.02 | .907 | | | | .970 |
| Occipital_Mid_L | | .11 | .561 | | | | .856 |
| Occipital_Mid_R | | .18 | .327 | | | | .765 |
| Occipital_Sup_L | | -.16 | .379 | | | | .800 |
| Occipital_Sup_R | | -.07 | .694 | | | | .915 |
| Olfactory_L | | .13 | .494 | | | | .856 |
| Olfactory_R | | .21 | .244 | | | | .765 |
| Pallidum_L | | -.04 | .839 | | | | .957 |
| Pallidum_R | | .02 | .909 | | | | .970 |
| Paracentral_Lobule_L | | .20 | .271 | | | | .765 |
| Paracentral_Lobule_R | | .06 | .744 | | | | .918 |
| ParaHippocampal_L | | -.04 | .821 | | | | .951 |
| ParaHippocampal_R | | .14 | .444 | | | | .851 |
| Parietal_Inf_L | | .00 | .981 | | | | .991 |
| Parietal_Inf_R | | .13 | .492 | | | | .856 |
| Parietal_Sup_L | | -.07 | .710 | | | | .915 |
| Parietal_Sup_R | | -.04 | .810 | | | | .951 |
| Postcentral_L | | .01 | .937 | | | | .977 |
| Postcentral_R | | .10 | .600 | | | | .864 |
| Precentral_L | | -.12 | .512 | | | | .856 |
| Precentral_R | | -.10 | .585 | | | | .856 |
| Precuneus_L | | .04 | .813 | | | | .951 |
| Precuneus_R | | .12 | .530 | | | | .856 |
| Putamen_L | | .00 | .997 | | | | .997 |
| Putamen_R | | -.04 | .846 | | | | .957 |
| Rectus_L | | .29 | .108 | | | | .747 |
| Rectus_R | | .19 | .289 | | | | .765 |
| Rolandic_Oper_L | | .02 | .906 | | | | .970 |
| Rolandic_Oper_R | | -.09 | .635 | | | | .897 |
| Supp_Motor_Area_L | | .24 | .181 | | | | .765 |
| Supp_Motor_Area_R | | .18 | .330 | | | | .765 |
| SupraMarginal_L | | .17 | .338 | | | | .765 |
| SupraMarginal_R | | .11 | .540 | | | | .856 |
| Temporal_Inf_L | | .08 | .652 | | | | .898 |
| Temporal_Inf_R | | -.02 | .901 | | | | .970 |
| Temporal_Mid_L | | -.21 | .243 | | | | .765 |
| Temporal_Mid_R | | -.21 | .259 | | | | .765 |
| Temporal_Pole_Mid_L | | .33 | .069 | | | | .656 |
| Temporal_Pole_Mid_R | | .38 | .031* | | | | .644 |
| Temporal_Pole_Sup_L | | .24 | .180 | | | | .765 |
| Temporal_Pole_Sup_R | | .29 | .110 | | | | .747 |
| Temporal_Sup_L | | -.17 | .350 | | | | .773 |
| Temporal_Sup_R | | .02 | .919 | | | | .970 |
| Thalamus_L | | .20 | .284 | | | | .765 |
| Thalamus_R | | .07 | .713 | | | | .915 |
| *Association significant at the uncorrected level p < .05  ** Association significant at the fdr corrected level q <.05 | |  |  | | | |  |

S5 Table. Correlations between the Regional Nodal Degree measures for 92 brain regions and the total items of the ADHD-RS IV scores of children and adolescents with ADHD combined and ADHD inattentive types.

| ADHD Combined and ADHD Inattentive Type Participants (*n* = 34) | | | | | |
| --- | --- | --- | --- | --- | --- |
|  | *r^2^* | | | *p* | *q* |
| Amygdala_L | | -0.01 | 0.963 | | 0.971 |
| Amygdala_R | | 0.09 | 0.63 | | 0.797 |
| Angular_L | | -0.15 | 0.427 | | 0.78 |
| Angular_R | | -0.01 | 0.971 | | 0.971 |
| Calcarine_L | | -0.12 | 0.515 | | 0.797 |
| Calcarine_R | | 0.04 | 0.809 | | 0.873 |
| Caudate_L | | -0.24 | 0.195 | | 0.662 |
| Caudate_R | | -0.27 | 0.134 | | 0.662 |
| Cerebellum_L | | -0.35 | 0.047* | | 0.515 |
| Cerebellum_R | | -0.02 | 0.931 | | 0.971 |
| Cingulum_Ant_L | | -0.14 | 0.448 | | 0.795 |
| Cingulum_Ant_R | | -0.17 | 0.358 | | 0.718 |
| Cingulum_Mid_L | | -0.38 | 0.032* | | 0.515 |
| Cingulum_Mid_R | | -0.28 | 0.127 | | 0.662 |
| Cingulum_Post_L | | -0.3 | 0.096 | | 0.608 |
| Cingulum_Post_R | | -0.2 | 0.272 | | 0.691 |
| Cuneus_L | | -0.46 | 0.008* | | 0.515 |
| Cuneus_R | | -0.34 | 0.058 | | 0.515 |
| Frontal_Inf_Oper_L | | -0.2 | 0.282 | | 0.691 |
| Frontal_Inf_Oper_R | | -0.23 | 0.205 | | 0.662 |
| Frontal_Inf_Orb_L | | 0.2 | 0.272 | | 0.691 |
| Frontal_Inf_Orb_R | | 0.32 | 0.074 | | 0.541 |
| Frontal_Inf_Tri_L | | 0.24 | 0.177 | | 0.662 |
| Frontal_Inf_Tri_R | | 0.41 | 0.019* | | 0.515 |
| Frontal_Med_Orb_L | | 0.1 | 0.602 | | 0.797 |
| Frontal_Med_Orb_R | | 0.33 | 0.062 | | 0.515 |
| Frontal_Mid_L | | -0.4 | 0.024* | | 0.515 |
| Frontal_Mid_Orb_L | | -0.29 | 0.11 | | 0.653 |
| Frontal_Mid_Orb_R | | -0.06 | 0.759 | | 0.851 |
| Frontal_Mid_R | | -0.08 | 0.663 | | 0.797 |
| Frontal_Sup_L | | -0.39 | 0.029* | | 0.515 |
| Frontal_Sup_Medial_L | | -0.35 | 0.053 | | 0.515 |
| Frontal_Sup_Medial_R | | -0.25 | 0.171 | | 0.662 |
| Frontal_Sup_Orb_L | | -0.1 | 0.582 | | 0.797 |
| Frontal_Sup_Orb_R | | 0.21 | 0.247 | | 0.691 |
| Frontal_Sup_R | | 0.24 | 0.186 | | 0.662 |
| Fusiform_L | | -0.33 | 0.065 | | 0.515 |
| Fusiform_R | | 0.18 | 0.32 | | 0.691 |
| Heschl_L | | 0.2 | 0.267 | | 0.691 |
| Heschl_R | | -0.1 | 0.596 | | 0.797 |
| Hippocampus_L | | 0.16 | 0.395 | | 0.75 |
| Hippocampus_R | | -0.08 | 0.655 | | 0.797 |
| Insula_R | | 0.23 | 0.213 | | 0.662 |
| Insula_L | | -0.03 | 0.858 | | 0.916 |
| Lingual_L | | -0.26 | 0.151 | | 0.662 |
| Lingual_R | | -0.19 | 0.3 | | 0.691 |
| Occipital_Inf_L | | 0.12 | 0.53 | | 0.797 |
| Occipital_Inf_R | | -0.12 | 0.497 | | 0.797 |
| Occipital_Mid_L | | 0.1 | 0.572 | | 0.797 |
| Occipital_Mid_R | | 0.09 | 0.643 | | 0.797 |
| Occipital_Sup_L | | -0.19 | 0.303 | | 0.691 |
| Occipital_Sup_R | | -0.14 | 0.46 | | 0.795 |
| Olfactory_L | | 0.01 | 0.968 | | 0.971 |
| Olfactory_R | | 0.22 | 0.223 | | 0.662 |
| Pallidum_L | | -0.17 | 0.363 | | 0.718 |
| Pallidum_R | | -0.05 | 0.798 | | 0.871 |
| Paracentral_Lobule_L | | 0.31 | 0.086 | | 0.584 |
| Paracentral_Lobule_R | | 0.16 | 0.392 | | 0.75 |
| ParaHippocampal_L | | -0.09 | 0.627 | | 0.797 |
| ParaHippocampal_R | | 0.11 | 0.532 | | 0.797 |
| Parietal_Inf_L | | -0.15 | 0.412 | | 0.767 |
| Parietal_Inf_R | | 0.07 | 0.714 | | 0.848 |
| Parietal_Sup_L | | -0.06 | 0.761 | | 0.851 |
| Parietal_Sup_R | | -0.06 | 0.737 | | 0.851 |
| Postcentral_L | | 0.06 | 0.755 | | 0.851 |
| Postcentral_R | | 0.2 | 0.278 | | 0.691 |
| Precentral_L | | -0.17 | 0.341 | | 0.718 |
| Precentral_R | | -0.09 | 0.641 | | 0.797 |
| Precuneus_L | | 0.09 | 0.608 | | 0.797 |
| Precuneus_R | | 0.17 | 0.349 | | 0.718 |
| Putamen_L | | 0.18 | 0.318 | | 0.691 |
| Putamen_R | | 0.08 | 0.652 | | 0.797 |
| Rectus_L | | 0.35 | 0.052 | | 0.515 |
| Rectus_R | | 0.27 | 0.136 | | 0.662 |
| Rolandic_Oper_L | | 0.18 | 0.32 | | 0.691 |
| Rolandic_Oper_R | | 0.08 | 0.645 | | 0.797 |
| Supp_Motor_Area_L | | 0.24 | 0.182 | | 0.662 |
| Supp_Motor_Area_R | | 0.23 | 0.214 | | 0.662 |
| SupraMarginal_L | | 0.22 | 0.221 | | 0.662 |
| SupraMarginal_R | | 0.14 | 0.46 | | 0.795 |
| Temporal_Inf_L | | 0.12 | 0.496 | | 0.797 |
| Temporal_Inf_R | | 0.01 | 0.954 | | 0.971 |
| Temporal_Mid_L | | 0.01 | 0.957 | | 0.971 |
| Temporal_Mid_R | | -0.13 | 0.491 | | 0.797 |
| Temporal_Pole_Mid_L | | 0.36 | 0.046* | | 0.515 |
| Temporal_Pole_Mid_R | | 0.24 | 0.179 | | 0.662 |
| Temporal_Pole_Sup_L | | 0.12 | 0.512 | | 0.797 |
| Temporal_Pole_Sup_R | | 0.19 | 0.295 | | 0.691 |
| Temporal_Sup_L | | 0.05 | 0.781 | | 0.863 |
| Temporal_Sup_R | | 0.09 | 0.639 | | 0.797 |
| Thalamus_L | | 0.23 | 0.204 | | 0.662 |
| Thalamus_R | | 0.12 | 0.505 | | 0.797 |
